# Pyogenic brain abscesses in Qatar: A national retrospective observational study

**DOI:** 10.1016/j.ijregi.2026.101035

**Published:** 2026-10-05

**Authors:** Sabeen Zara, Sara Al Balushi, Junais Koleri, Afia Aziz, Soubiya Ansari, Mu'ath Hussein, Aseel H. Alzibdeh, Ahmed Saleh, Waqar Munir, Ali S. Omrani, Muna Al Maslamani

**Affiliations:** 1 Division of Infectious Diseases, Department of Internal Medicine, Communicable Disease Center, Hamad Medical Corporation, Doha, Qatar; 2 Department of Internal Medicine, Hamad Medical Corporation, Doha, Qatar; 3 Department of Neurosurgery, Hamad Medical Corporation, Doha, Qatar; 4 Qatar University College of Medicine, Doha, Qatar

**Keywords:** Pyogenic brain abscess, Intracranial infection, Neurosurgical intervention, Qatar

## Abstract

**Objectives:**

Pyogenic brain abscess is a life-threatening intracranial infection requiring timely diagnosis and coordinated medical–surgical management. Regional data from the Gulf area remains scarce.

**Methods:**

We retrospectively analyzed demographic, clinical, microbiological, management, and outcome data from adults diagnosed with pyogenic brain abscess based on clinical presentation and neuroimaging findings at Hamad Medical Corporation, Qatar, between 2015 and 2023. Outcomes were categorized using the Glasgow Outcome Scale (GOS) as favorable (GOS 4-5) or unfavorable (GOS 1-3).

**Results:**

Forty-eight patients were included; 41/48 (85%) were male, with a median age of 34 years. Common comorbidities and predisposing conditions included diabetes mellitus, otitis/mastoiditis, and sinusitis. A microbiological diagnosis was established in 35/48 (73%) patients. The most frequently isolated pathogens were the *Streptococcus anginosus* group*, Staphylococcus aureus,* and *Klebsiella pneumoniae.* Surgical intervention was performed in 38/48 (79%) patients, and 35/48 (73%) achieved a favorable outcome. In unadjusted analyses, recent neurosurgical procedures and Gram-negative infections were more frequent among patients with unfavorable outcomes. These patients had lower baseline Glasgow Coma Scale scores and more frequently presented with altered consciousness.

**Conclusion:**

In unadjusted analyses, favorable outcomes were more frequent among patients who underwent surgical intervention and those presenting with preserved consciousness. However, causality cannot be inferred.

## Highlights

• Microbiological diagnosis was established in 73% of patients.
• The leading pathogens were *Streptococcus anginosus* group*, Staphylococcus aureus*, and *Klebsiella pneumoniae.*
• Neurosurgery was performed in 79%, and 73% achieved favorable outcomes.
• Lower Glasgow Coma Scale scores and altered consciousness accompanied unfavorable outcomes.
• Recent neurosurgery and Gram-negative organisms appeared more frequent in unfavorable outcomes.

## Graphical abstract

**Figure:**
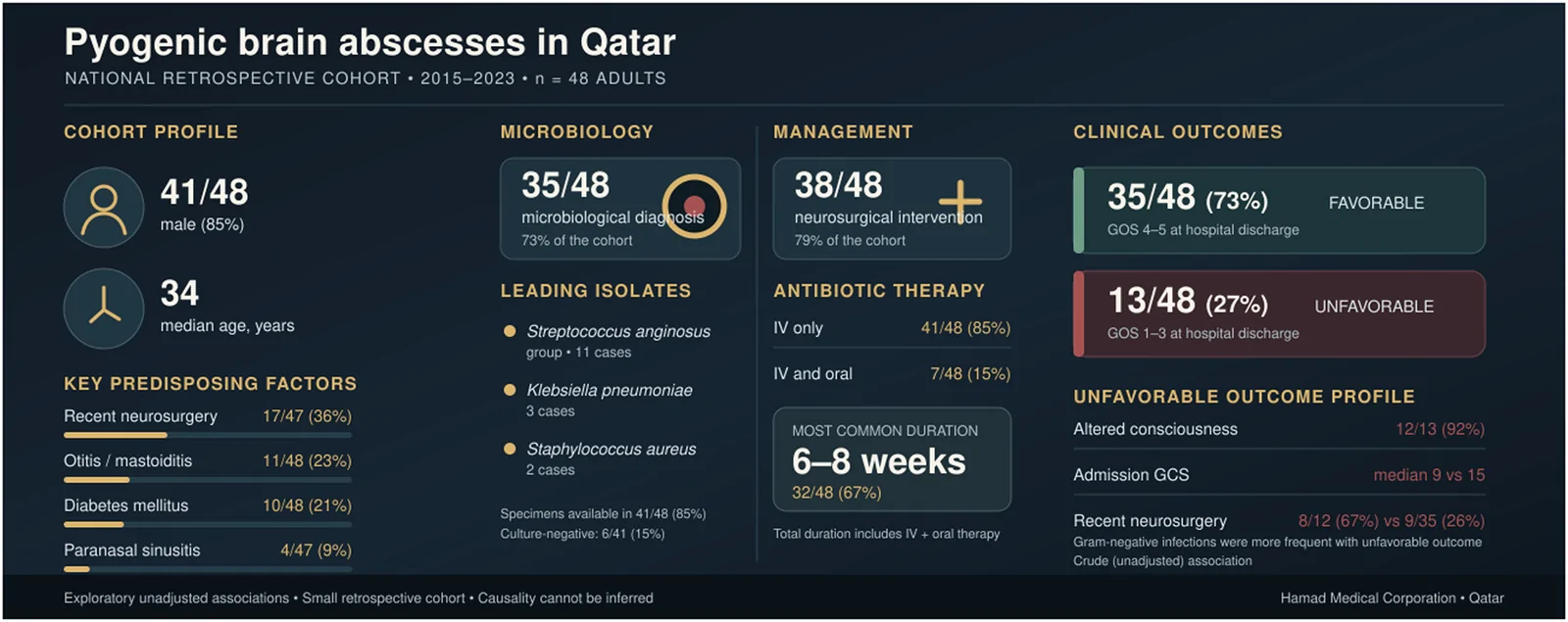

## Introduction

Pyogenic brain abscess is a life-threatening, focal, suppurative intracranial infection that remains a neurological emergency despite recent advances in neurocritical care [1,2]. Risk factors for pyogenic brain abscesses include immunosuppression, chronic otitis media or paranasal sinus infection, congenital heart disease, diabetes, and cranial trauma or neurosurgical procedures [3,4].

Clinical outcomes in patients with pyogenic brain abscesses depend on timely diagnosis, effective antibiotic therapy, and surgical management, when indicated [1,5]. Prognosis has improved in recent decades, with mortality reported at approximately 10-20% in contemporary series [1,2,6,7]. However, mortality remains elevated in older patients and in those with comorbidities, including malignancy, immunosuppression, and systemic infection [8].

Qatar is a rapidly developing nation with a relatively young, predominantly male, and ethnically diverse expatriate workforce, potentially influencing disease epidemiology, clinical characteristics, and outcomes. Published national and regional data on pyogenic brain abscesses are lacking, and to our knowledge, this is the first study from Qatar to address this condition.

The primary objective of this study was to describe the demographic, clinical, microbiological, and management characteristics of adult pyogenic brain abscess in Qatar’s national public hospital system. A secondary exploratory objective was to identify factors associated with unfavorable in-hospital outcomes.

## Methods

### Study design and population

This was a retrospective exploratory observational cohort study including all adult patients (≥18 years) with a diagnosis of pyogenic brain abscess admitted to tertiary care hospitals under Hamad Medical Corporation, Qatar, between January 2015 and June 2023.

Using electronic health records, neuroimaging reports, and the microbiology database, we retrospectively included all patients aged ≥18 years with clinical and radiological features consistent with pyogenic brain abscess, with or without microbiological confirmation. Patients diagnosed with tuberculoma or neurocysticercosis without pyogenic brain abscess were excluded.

### Data collection

Demographic, clinical, laboratory, radiological, microbiological, treatment, and outcome data were extracted from patient records using a standardized data collection form. Variables included age, sex, nationality, comorbidities, neurological status, presenting symptoms, laboratory findings, abscess location, documented abscess rupture, surgical intervention, microbiological results (culture and direct microscopy), and antibiotic regimens.

Otogenic and sinonasal infectious foci were determined from compatible imaging findings and documented ear, nose, and throat assessment.

Functional outcome at hospital discharge was retrospectively classified using the Glasgow Outcome Scale (GOS) based on the documented neurological and functional status at discharge. Scores of 4-5 were classified as favorable, while scores of 1-3 were classified as unfavorable. Empiric antibiotic therapy was defined as the initial broad-spectrum intravenous antibiotics started before microbiological confirmation; definitive therapy was defined as any antibiotic adjustment based on culture and susceptibility results.

### Statistical analysis

Continuous variables were reported as medians (interquartile ranges [IQRs]) and compared using the Mann-Whitney *U* test, except for mean arterial pressure, which was reported as the mean (standard deviation) and compared using an independent-samples *t*-test. Categorical variables were summarized as counts and percentages and compared using chi-square or Fisher exact test, as appropriate. Based on the limited number of unfavorable outcome events, only univariable comparisons were performed, and no multivariable regression analysis was undertaken. All analyses were performed using Stata/SE version 14.2 (StataCorp, College Station, TX, USA). Missing data were handled using complete-case analysis for each variable, and the corresponding denominators were reported. No adjustment was made for multiple comparisons because the analyses were exploratory. All reported associations are unadjusted and should therefore be interpreted cautiously.

## Results

### Demographics and clinical characteristics

A total of 48 patients with confirmed brain abscesses were included in the study. Of these, 35/48 (73%) had a favorable outcome and 13/48 (27%) had an unfavorable outcome. The median age at diagnosis was 32 years (IQR = 27-41) in the favorable group and 37 years (IQR = 29-48) in the unfavorable group. Overall, 41/48 (85%) of patients were male, with a higher proportion in the favorable group compared with the unfavorable group (33/35 [94%] vs 8/13 [62%]).

The most frequent symptoms in the favorable outcome group were headache (26/35 [74%]), neurological deficit (14/35 [40%]), and fever (15/35 [43%]). In contrast, patients with unfavorable outcomes most commonly presented with altered level of consciousness (12/13 [92%]; *P* < 0.001), neurological deficit (11/13 [85%]; *P* = 0.006), and fever (8/13 [62%]). The GCS score at admission was significantly lower in the unfavorable outcome group (median 9, IQR = 5-14) than in the favorable outcome group (median 15, IQR = 15-15; *P* < 0.001). Additional baseline characteristics are presented in Table 1.

**Table 1:** Baseline demographic and clinical characteristics of patients with brain abscess, stratified by outcome group.

Variable	Entire cohort	Unfavorable outcomes	Favorable outcomes
	*N* = 48	*N* = 13	*N* = 35
**Demographics**			
Age, years^a^	34 (27-48)	37 (29-48)	32 (27-41)
Male sex (%)	41/48 (85)	8/13 (62)	33/35 (94)
**Signs and symptoms (%)**			
Headache	30/48 (63)	4/13 (31)	26/35 (74)
Fever ≥38°C	23/48 (48)	8/13 (62)	15/35 (43)
Nausea/vomiting	15/48 (31)	4/13 (31)	11/35 (31)
Seizure	15/48 (31)	5/13 (38)	10/35 (29)
Neurological deficit	25/48 (52)	11/13 (85)	14/35 (40)
Altered level of consciousness	22/48 (46)	12/13 (92)	10/35 (29)
Signs of meningeal irritation	4/48 (8)	1/13 (8)	3/35 (9)
**Blood tests (%)**			
Leukocytosis >10 × 10^9^/l	37/48 (77)	10/13 (77)	27/35 (77)
Blood cultures	40/48 (83)	13/13 (100)	27/35 (77)
Positive blood culture^b^	7/40 (18)	4/13 (31)	3/27 (11)
**CSF findings (%)**			
Lumbar puncture	10/48 (21)	4/13 (31)	6/35 (17)
Positive CSF culture^b^	4/10 (40)	3/4 (75)	1/6 (17)
CSF pleocytosis >5 cells/µL^b^	8/10 (80)	4/4 (100)	4/6 (67)
CSF protein >0.45 g/l b	7/10 (70)	4/4 (100)	3/6 (50)
**Neuroimaging findings (%)**			
Single abscess	39/48 (81)	11/13 (85)	28/35 (80)
Multiple abscesses	9/48 (19)	2/13 (15)	7/35 (20)
**Abscess location (%)**			
Frontal lobe only	11/48 (23)	3/13 (23)	8/35 (23)
Parietal lobe only	7/48 (15)	2/13 (15)	5/35 (14)
Temporal lobe only	11/48 (23)	3/13 (23)	8/35 (23)
Occipital lobe only	7/48 (15)	0/13 (0)	7/35 (20)
Multilobe involvement	12/48 (25)	5/13 (38)	7/35 (20)
**Microorganism (%)**			
Mixed	9/41 (22)	2/10 (20)	7/31 (23)
No growth	6/41 (15)	0/10 (0)	6/31 (19)
Fungal	1/41 (2)	1/10 (10)	0/31 (0)
Gram-negative bacteria	8/41 (20)	5/10 (50)	3/31 (10)
Gram-positive bacteria	17/41 (41)	2/10 (20)	15/31 (48)
No specimen available for microbiological analysis	7/48 (15)	3/13 (23)	4/35 (11)
**Intervention (%)**			
Surgery	38/48 (79)	7/13 (54)	31/35 (89)
Drainage	19/38 (50)	3/7 (43)	16/31 (52)
Evacuation	4/38 (11)	0/7 (0)	4/31 (13)
Resection	15/38 (39)	4/7 (57)	11/31 (35)
Dexamethasone	19/48 (40)	5/13 (38)	14/35 (40)
Antibiotics (IV/oral)			
IV only	41/48 (85)	12/13 (92)	29/35 (83)
IV and oral	7/48 (15)	1/13 (8)	6/35 (17)
**Duration of antibiotics (%)**			
<6 weeks	10/48 (21)	4/13 (31)	6/35 (17)
6-8 weeks	32/48 (67)	5/13 (38)	27/35 (77)
>8 weeks	6/48 (13)	4/13 (31)	2/35 (6)
**SOFA score**			
P/F ratio (mmHg)^a^	475 (404-520)	404 (240-485)	490 (450-525)
Missing (%)	14/48 (29)	2/13 (15)	12/35 (34)
GCS^a^	15 (14-15)	9 (5-14)	15 (15-15)
Dopamine (yes, %)	2/46 (4)	1/11 (9)	1/35 (3)
Missing (%)	2/48 (4)	2/13 (15)	0/35 (0)
Bilirubin, µmol/l^a^	9 (6-13)	8 (5-11)	9 (8-16)
MAP, mmHg^c^	89 (19)	82 (15)	92 (20)
Missing (%)	2/48 (4)	1/13 (8)	1/35 (3)
Creatinine, µmol/l^a^	75 (61-82)	61 (50-74)	76 (68-86)
Platelets × 10⁹/l^a^	275 (200-358)	275 (182-439)	273 (198-325)
Missing (%)	1/48 (2)	0/13 (0)	1/35 (3)
**Predisposing factors/comorbidities**			
Paranasal sinusitis (%)	4/47 (9)	0/12 (0)	4/35 (11)
Missing (%)	1/48 (2)	1/13 (8)	0/35 (0)
Dental infection (%)	3/48 (6)	0/13 (0)	3/35 (9)
Chronic otitis media or mastoiditis (%)	11/48 (23)	2/13 (15)	9/35 (26)
Pneumonia (%)	2/48 (4)	2/13 (15)	0/35 (0)
Bacterial endocarditis (%)	0/48 (0)	0/13 (0)	0/35 (0)
Cyanotic heart disease (%)	0/48 (0)	0/13 (0)	0/35 (0)
Pre-existing hemiplegia (%)	3/48 (6)	2/13 (15)	1/35 (3)
Recent neurosurgical procedure (%)	17/47 (36)	8/12 (67)	9/35 (26)
Missing (%)	1/48 (2)	1/13 (8)	0/35 (0)
Cranial trauma (%)	7/48 (15)	2/13 (15)	5/35 (14)
Diabetes (%)	10/48 (21)	3/13 (23)	7/35 (20)
Cancer, leukemia, or lymphoma (%)	3/48 (6)	2/13 (15)	1/35 (3)
Liver cirrhosis (%)	0/48 (0)	0/13 (0)	0/35 (0)
Prior stroke (%)	4/48 (8)	4/13 (31)	0/35 (0)
Granulocytopenia	2/47 (4)	2/13 (15)	0/34 (0)
Missing (%)	1/48 (2)	0/13 (0)	1/35 (3)

Note: Categorical data are presented as *n*/*N* (%). Continuous variables are presented as the median (interquartile range) or the mean (standard deviation), as indicated. Denominators vary because of missing data or subgroup analyses.

a Median (interquartile range).

b Subgroup analysis.

c Mean (standard deviation). Abbreviations: CSF, cerebrospinal fluid; GCS, Glasgow Coma Scale; IV, intravenous; MAP, mean arterial pressure; P/F ratio, ratio of arterial oxygen partial pressure to fraction of inspired oxygen; SOFA, Sequential Organ Failure Assessment.

### Predisposing factors and comorbidities

Overall, the most common underlying conditions were chronic otitis media or mastoiditis (11/48 [23%]), diabetes mellitus (10/48 [21%]), and paranasal sinusitis (4/47 [9%]). Prior stroke occurred in 4/48 (8%) patients. A recent neurosurgical procedure was the leading predisposing factor (17/47 [36%]), followed by cranial trauma (7/48 [15%]) and dental infection (3/48 [6%]).

By outcome, diabetes was similarly distributed between the favorable (7/35 [20%]) and unfavorable (3/13 [23%]) outcome groups. A recent neurosurgical procedure was more frequent in the unfavorable group (8/12 [67%] vs 9/35 [26%]), and all prior strokes occurred in patients with unfavorable outcomes (4/13 [31%] vs 0/35 [0%]). Additional underlying conditions and predisposing factors are presented in Table 1.

### Laboratory results

Leukocytosis (>10 × 10⁹/l) was present in 27/35 (77%) patients in the favorable outcome group and 10/13 (77%) in the unfavorable outcome group. Blood cultures were obtained in 40/48 (83%) patients, yielding positive results in 3/27 (11%) patients in the favorable outcome group and 4/13 (31%) in the unfavorable outcome group. Lumbar puncture was performed in 10/48 (21%) patients, including 6/35 (17%) in the favorable outcome group and 4/13 (31%) in the unfavorable outcome group. Among those who underwent cerebrospinal fluid (CSF) analysis, pleocytosis (>5 cells/µL) was observed in 4/6 (67%) patients in the favorable outcome group and 4/4 (100%) in the unfavorable outcome group. Elevated CSF protein (>0.45 g/l) was present in 3/6 (50%) patients in the favorable outcome group and 4/4 (100%) in the unfavorable outcome group. CSF cultures were positive in 4/10 (40%) patients overall, including 1/6 (17%) in the favorable outcome group and 3/4 (75%) in the unfavorable outcome group (Table 1).

### Neuroimaging findings

All patients underwent neuroimaging with computed tomography, magnetic resonance imaging (MRI), or both. A single abscess was identified in 39/48 (81%) patients, including 28/35 (80%) in the favorable outcome group and 11/13 (85%) in the unfavorable outcome group. Multiple abscesses were present in 9/48 (19%) patients, including 7/35 (20%) in the favorable outcome group and 2/13 (15%) in the unfavorable outcome group. The most frequent locations in the favorable outcome group were the frontal lobe (8/35 [23%]), temporal lobe (8/35 [23%]), occipital lobe (7/35 [20%]), and parietal lobe (5/35 [14%]). In the unfavorable outcome group, abscesses were most commonly located in the frontal lobe (3/13 [23%]), temporal lobe (3/13 [23%]), and parietal lobe (2/13 [15%]), with no cases involving the occipital lobe. Multilobar involvement was noted in 7/35 (20%) patients in the favorable outcome group and 5/13 (38%) in the unfavorable outcome group (Table 1). No brain abscess rupture was documented in the available medical or radiological records; therefore, its association with CSF culture positivity and clinical outcome could not be assessed.

### Microbiology

Microbiological specimens were available for 41/48 (85%) patients. Of the 10 patients who did not undergo abscess drainage, evacuation, or resection, three had diagnostic specimens available, while no microbiological specimen was available for the remaining 7/48 (15%) patients. Culture of brain biopsy tissue grew *Aspergillus flavus* in one patient, bronchoalveolar lavage grew *Nocardia* species in a patient with disseminated nocardiosis, and CSF obtained through an external ventricular drain grew a Gram-negative organism in one patient. A causative microorganism was identified in 35/41 (85%) patients, predominantly from operative cultures, while 6/41 (15%) yielded no growth. In the favorable outcome group, Gram-positive bacteria were most common (15/31 [48%]), with the *Streptococcus anginosus* group being the predominant isolate (11/15 [73%]; Table 2). Gram-negative organisms were identified in 3/31 patients (10%), and mixed infections in 7/31 patients (23%). In contrast, Gram-negative organisms were most common in the unfavorable outcome group (5/10 [50%]), most commonly *Klebsiella* species (3/5 [60%]). Mixed infections were present in 2/10 patients (20%), while Gram-positive organisms accounted for 2/10 patients (20%). The overall distribution of mixed infections, fungal isolates, Gram-positive and Gram-negative bacteria, and culture-negative cases is presented in Table 1, while selected individual pathogens and associated outcomes are summarized in Table 2, Table 3.

**Table 2:** Selected microbiological findings and associated clinical outcomes.

Organism group	Total	Favorable outcome (%)	Unfavorable outcome (%)
*Streptococcus anginosus* group	11	11/11 (100)	0/11 (0)
*Staphylococcus aureus* (MSSA)	2	2/2 (100)	0/2 (0)
*Klebsiella pneumoniae*	3	0/3 (0)	3/3 (100)
Mixed aerobic–anaerobic infections	9	7/9 (78)	2/9 (22)
Culture-negative	6	6/6 (100)	0/6 (0)
Rare pathogens (*Nocardia species/Aspergillus flavus*)	3	1/3 (33)	2/3 (67)

Abbreviation: MSSA, methicillin-susceptible *Staphylococcus aureus*.

**Table 3:** Clinical characteristics, risk factors, and outcomes of rare pathogen infections.

Pathogen	Age, years	Sex	Nationality	Predisposing factors	Location	Diagnosis (culture sample)	Outcome
***Burkholderia pseudomallei***	59	M	Indian	Previous neurosurgical procedure	Temporo­parietal abscess	Tissue culture (abscess evacuation)	Favorable
***Nocardia* species**	50	F	Pakistani	Renal transplant recipient receiving immuno­suppression	Frontal lobe abscess	Pus culture (neurosurgical resection)	Favorable
***Nocardia* species**	29	F	Filipino	SLE; immunosuppressive therapy, disseminated nocardiosis	Temporal lobe with multilobar involvement	BAL culture	Unfavorable (required long-term care)
***Aspergillus flavus***	32	F	Ethiopian	SLE; immunosuppressive therapy	Multilobar abscesses	Brain biopsy culture	Unfavorable (death within 1 week)

Abbreviations: BAL, bronchoalveolar lavage; F, female; M, male; SLE, systemic lupus erythematosus.

### Treatment and outcomes

Antimicrobial therapy was initiated empirically in all patients and adjusted according to microbiological results, where available. The most common regimens were ceftriaxone plus metronidazole, with or without vancomycin, for community-acquired infections, and meropenem, vancomycin, or linezolid for postsurgical infections or when multidrug-resistant organisms were suspected. Overall, 41/48 (85%) patients received exclusively intravenous antibiotics, while 7/48 (15%) received both intravenous and oral therapy. The use of exclusively intravenous therapy was similar between the unfavorable and favorable outcome groups (12/13 [92%] vs 29/35 [83%]).

The duration of antibiotic therapy was less than 6 weeks in 10/48 (21%) patients, including 6/35 (17%) in the favorable outcome group and 4/13 (31%) in the unfavorable outcome group. Treatment lasted 6-8 weeks in 32/48 (67%) patients, including 27/35 (77%) in the favorable outcome group and 5/13 (38%) in the unfavorable outcome group. Treatment exceeding 8 weeks was given to 6/48 (13%) patients, including 2/35 (6%) in the favorable outcome group and 4/13 (31%) in the unfavorable outcome group. Dexamethasone was administered to 19/48 (40%) patients, with similar use in the unfavorable and favorable outcome groups (5/13 [38%] vs 14/35 [40%]).

Neurosurgical intervention consisting of abscess drainage, evacuation, or resection was performed in 38/48 (79%) patients. The procedures included drainage in 19/38 (50%), evacuation in 4/38 (11%), and resection in 15/38 (39%) patients. Neurosurgical intervention was more frequent in the favorable outcome group (31/35 [89%]) than in the unfavorable outcome group (7/13 [54%]; *P* = 0.016).

## Discussion

To our knowledge, this study provides the first national descriptive cohort of adult pyogenic brain abscess in Qatar and generates hypotheses regarding outcome-associated clinical and microbiological features that warrant validation in larger multicenter studies.

Our study confirms that pyogenic brain abscess remains a disease of young adults (median age, 34 years), a pattern consistently reported from North America, Europe, and East Asia [2,7,8]. The male predominance in our cohort (41/48 [85%]) is consistent with the approximately 2:1 male predominance generally reported in brain abscess cohorts and may have been further influenced by Qatar’s predominantly male expatriate population [2,7]^.^ Cranial trauma was documented in 7/48 (15%) patients, two of whom had also undergone a recent neurosurgical procedure. No occupational association was documented in the available records. Common predisposing factors were diabetes, chronic ear or sinus disease, and postsurgical or posttraumatic states. The high proportion of recent neurosurgical procedures (17/47 [36%]) underscores the importance of neurosurgical and healthcare-associated factors in contemporary practice [9]_._

Clinical presentation was typical of brain abscess series: headache, focal neurologic deficit, seizures, and altered mental status [[1], [2], [3],^7^]. In unadjusted analyses, altered consciousness and lower GCS scores at admission were associated with unfavorable outcomes in our cohort, consistent with previous reports [5,6,10].

The *S. anginosus* group and *S. aureus* predominated, with notable representation of *Klebsiella* species, *Pseudomonas* species*,* and Enterobacterales*,* especially in postsurgical patients and patients with diabetes. In unadjusted analyses, Gram-negative infections appeared to be associated with unfavorable outcomes, consistent with previous reports [9,10]. However, Gram-negative organisms were more frequently encountered in postsurgical or healthcare-associated settings. Differences in the underlying clinical setting and baseline prognosis, together with the small number of monomicrobial Gram-negative infections, may therefore have contributed to the observed association. Although based on small numbers, some exploratory patterns were observed: abscesses caused by *S. anginosus* group, often originating from oral, sinus, or pleural infections, were associated with a favorable outcome in all 11 patients in our series, consistent with their typically indolent nature and responsiveness to antibiotics [2,7]. In contrast, all four patients with *Klebsiella* species or *Escherichia coli* infection had unfavorable outcomes, consistent with previous reports [11,12]. Seven of nine patients (78%) with mixed aerobic–anaerobic infections had favorable outcomes. Both patients with *S. aureus* infection and all six patients with culture-negative results had favorable outcomes. The three patients with rare pathogens (*Nocardia* species or *Aspergillus* species) were immunocompromised, and two had unfavorable outcomes.

Notably, we identified *Burkholderia pseudomallei* (‘melioidosis’) in an Indian patient, consistent with a possible imported infection. This finding aligns with previous reports documenting *B. pseudomallei* infection in endemic regions and among travelers or migrants [13,14]. Rare presentations included *Nocardia* species in a patient with systemic lupus erythematosus and a transplant recipient, as well as *A. flavus* in an immunosuppressed individual (Table 3). These cases underscore the importance of considering opportunistic pathogens in immunocompromised hosts. *Klebsiella pneumoniae* emerged as a frequent pathogen, particularly among South Asian patients and patients with diabetes, suggesting a possible association with regional and metabolic risk factors. However, the small number of cases precludes firm conclusions. This diversity in microbial etiology highlights the potential influence of host factors, such as immunosuppression, travel or migration history, and underlying comorbidities, in shaping the spectrum of pathogens responsible for brain abscesses.

Antibiotic selection in our cohort reflected these patterns, with broad-spectrum empiric regimens commonly used for postsurgical and health care-associated cases. The total duration of antibiotic therapy, including intravenous and oral treatment, was 6-8 weeks in 32/48 (67%) patients, less than 6 weeks in 10/48 (21%), and more than 8 weeks in 6/48 (13%). Among the 10 patients treated for less than 6 weeks, four underwent abscess resection; three completed 4 weeks of therapy, while one died before completing treatment. Three patients underwent drainage and had radiological resolution on follow-up MRI. Of the remaining three patients who did not undergo surgery, one had radiological resolution on follow-up MRI, while two died before completing therapy. The most frequently used duration of 6-8 weeks was consistent with the European Society of Clinical Microbiology and Infectious Diseases guidelines, which recommend 6-8 weeks of antibiotic therapy for aspirated or conservatively treated brain abscesses, while a shorter duration, such as 4 weeks, may be considered following excision [15]. Regimens were adapted to local susceptibility data, and postoperative abscesses often required broader-spectrum therapy based on microbiological findings and clinical context.

In unadjusted analyses, neurosurgical intervention, including drainage, evacuation, or resection, was associated with a favorable outcome (*P* = 0.016). Among the 38 patients who underwent neurosurgical intervention, 31/38 (82%) had a favorable outcome, compared with 4/10 (40%) patients managed with medical therapy alone. Similar associations have been reported previously [2,5,6]. However, this finding may be affected by confounding by indication and immortal time bias because patients selected for surgery may have differed in baseline prognosis and had to survive long enough to undergo intervention. Similar considerations apply to dexamethasone use; therefore, no causal treatment effect can be inferred.

These findings highlight the importance of rapid clinical recognition, timely neurosurgical assessment, and close monitoring, particularly among patients presenting with altered consciousness or those with postsurgical or health care-associated infections.

## Limitations

This study is limited by its retrospective design, relatively small sample size, and inclusion of patients from a single national healthcare system. The limited number of unfavorable outcomes precluded meaningful adjusted analyses. Consequently, all reported associations are crude (unadjusted) and susceptible to residual confounding and Type I error because of multiple comparisons. As GOS was retrospectively derived from documented neurological and functional status, some outcome misclassification is possible. Microbiological confirmation was not achieved in all cases, partly because of prior antibiotic exposure and limited specimen yield. Advanced molecular diagnostic techniques, including next-generation sequencing, may provide valuable adjuncts to conventional methods and improve pathogen detection in culture-negative cases [16].

## Conclusion

Pyogenic brain abscess remains an important and complex clinical challenge in Qatar. Our national cohort highlights diabetes, postsurgical infections, and the diversity of pathogens identified in this setting. In unadjusted analyses, preserved consciousness at presentation and neurosurgical intervention were associated with favorable outcomes. However, these associations do not establish causality and require validation in larger multicenter studies.

## Funding statement

This research did not receive any specific grant from funding agencies in the public, commercial, or not-for-profit sectors.

## Ethics approval

The study protocol was approved by the Institutional Review Board and Medical Research Center of Hamad Medical Corporation (MRC-01-22-455). The requirement for informed consent was waived because of the retrospective design and use of anonymized data.

## Data availability

The data supporting the findings of this study are available from the corresponding author upon reasonable request.

## CRediT authorship contribution statement

**Sabeen Zara:** Writing – review & editing, Writing – original draft, Investigation, Formal analysis, Data curation, Conceptualization. **Sara Al Balushi:** Formal analysis. **Junais Koleri:** Writing – review & editing. **Afia Aziz:** Data curation. **Soubiya Ansari:** Data curation. **Mu'ath Hussein:** Data curation. **Aseel H. Alzibdeh:** Writing – review & editing. **Ahmed Saleh:** Data curation. **Waqar Munir:** Supervision. **Ali S. Omrani:** Writing – review & editing, Supervision. **Muna Al Maslamani:** Supervision.

## Declaration of competing interest

The authors have no competing interests to declare.
